# Genomic Landscape of Thymic Carcinoma: A Large-Scale Analysis of Somatic Mutations, Demographic Disparities, and Metastatic Drivers from the AACR Project GENIE^®^ Cohort

**DOI:** 10.3390/cimb48010090

**Published:** 2026-01-16

**Authors:** Aden V. Chudziak, Tyson J. Morris, David Maliy, Grace S. Saglimbeni, Akaash Surendra, Beau Hsia, Huijun Li, Abubakar Tauseef

**Affiliations:** 1 School of Molecular Sciences, Arizona State University, Tempe, AZ 85281, USA; achudzia@asu.edu; 2 Department of Hematology and Oncology, Creighton University School of Medicine, Phoenix, AZ 85012, USA; tysonmorris@creighton.edu (T.J.M.); gracesaglimbeni@creighton.edu (G.S.S.); beauhsia@creighton.edu (B.H.); 3 College of Natural Sciences, Grand Canyon University, Phoenix, AZ 85017, USA; david.maliy@my.gcu.edu; 4 School of Life Sciences, Arizona State University, Tempe, AZ 85281, USA; asurend5@asu.edu; 5 Division of Hematology and Oncology, Department of Internal Medicine, Creighton University Medical Center, Omaha, NE 68124, USA; huijun.li@commonspirit.org

**Keywords:** thymic carcinoma, genomic landscape, somatic mutations, TP53, MTOR, AACR project GENIE, copy number alterations, metastasis, sex-specific mutations

## Abstract

Thymic carcinoma (TC) is a rare and aggressive malignancy with poor prognosis, and its genomic landscape remains incompletely defined. Identifying the somatic alterations that shape TC biology is essential for improving diagnostic precision, developing targeted therapies, and informing early detection strategies. We performed a retrospective genomic analysis of 141 TC tumor specimens from 134 patients using de-identified data from the American Association for Cancer Research (AACR) Project GENIE^®^ database. Somatic mutations and copy number alterations (CNAs) were characterized, and statistical analyses were conducted to evaluate associations with patient demographics (sex, race) and tumor site (primary vs. metastatic). The cohort was predominantly male (56.7%) and White (56.7%). The most frequently altered genes were TP53 (27.7%), CYLD (17.6%), and CDKN2A (12.1%). Recurrent homozygous deletions at chromosome 9p21.3 involving CDKN2A and CDKN2B were common. Sex-stratified analysis revealed several significant male-specific alterations. Although the Pacific Islander subgroup was small (*n* = 2), preliminary analysis suggested enrichment of alterations in key cancer-associated genes, including TP53, BRCA1, and STAT5B, underscoring the need for diverse representation in TC genomics. Notably, MTOR mutations were significantly enriched in a subset of local recurrences and lymph node metastases (*n* = 3; *q* = 0.013), suggesting a potential role in disease progression. This large-scale genomic analysis reinforces the central involvement of TP53, cell-cycle control, and chromatin-modifying pathways in TC. The identification of sex-associated and race-associated mutational patterns, together with the enrichment of MTOR alterations in recurrent and metastatic disease, highlights biologically plausible mechanisms of progression and potential therapeutic vulnerabilities. These findings support the value of comprehensive genomic profiling in TC and emphasize the need for prospective, multi-omic studies to validate these observations and guide the development of more personalized treatment strategies.

## 1. Introduction

Thymic carcinoma (TC) is a rare, aggressive neoplasm arising from thymic epithelial cells in the anterior (prevascular) mediastinum. Although accounting for a minority of thymic tumors, TC is typically invasive and often diagnosed at an advanced stage [1]. The World Health Organization (WHO) classifies TC as a malignant epithelial tumor of the thymus that lacks the organotypic features seen in thymomas and instead exhibits histological resemblance to carcinomas of other organs [2]. Clinically, patients may present with non-specific symptoms such as persistent cough, chest pain or pressure, dyspnea, hoarseness, or swelling of the face, neck, or upper body [2]. Pathologically, TC often demonstrates features such as necrosis, hemorrhage, infiltrative margins, and cystic degeneration—findings that can overlap with those seen in thymomas, necessitating histopathologic evaluation for definitive diagnosis [2]. Histologically, TC may display squamous differentiation, spindle cell morphology, and a prominent inflammatory infiltrate [2]. Prognosis is variable, with a reported five-year survival rate of approximately 30–50%, though outcomes are highly dependent on tumor histology and clinical stage at diagnosis [3]. TC is an exceptionally rare malignancy, representing less than 0.01% of all tumors [4]. Thymic epithelial tumors (TETs)—a group that includes both thymomas and thymic carcinomas—occur at an estimated incidence of 1.3 cases per million annually in the United States, totaling approximately 400 cases each year [5]. Of these, about 20% are classified as TC [3].

TC exhibits a higher prevalence in males, with a male-to-female ratio of approximately 1.5:1. While TC occurs across all racial groups, a higher incidence has been observed among individuals of Asian or Pacific Islander descent [6]. No specific lifestyle, inherited, or environmental risk factors have been identified for TC [7]. However, risk factors associated with other TETs, such as thymoma, include autoimmune conditions such as myasthenia gravis and pure red cell aplasia [8].

Recent genomic studies have identified *KIT* (CD117) as a potential oncogenic driver in TC, with *KIT* mutations detected in 19 of 22 patients in one cohort [9]. In addition to *KIT*, recurrent alterations in *TP53* and *CDKN2A* deletions are commonly associated with TC and may contribute to tumor progression. *CD5* has emerged as a valuable diagnostic biomarker, frequently expressed in TC but rarely in thymoma, aiding in histopathological differentiation [10]. TC is exceptionally rare, accounting for only 0.06% of all malignancies, and the limited sample sizes in most studies hinder the detection of low-frequency mutations and the establishment of molecular subtypes. Given that the 5-year survival rate of TC declines from 100% in stage I to 30–50% in stage IV, it is critical to develop effective screening methods [11,12,13]. Preliminary studies suggest that targeted screening programs in high-risk populations may enhance early diagnosis and improve overall survival rates [14].

Although progress has been made, the full range of genetic mutations associated with the development of TC is not fully understood [13]. Uncovering additional factors that contribute to tumor growth, resistance to treatment, and disease spread is vital for advancing diagnostic tools and therapeutic options. This study draws on a publicly accessible genomic database to provide a more comprehensive analysis of TC’s somatic mutation profile to support the development of improved therapies and early detection methods.

## 2. Materials and Methods

This study was exempt from institutional review board (IRB) approval by Creighton University (Phoenix, AZ, USA) because it utilized de-identified, publicly available data from the American Association for Cancer Research (AACR) Project GENIE^®^ (Genomics Evidence Neoplasia Information Exchange) database. Data were accessed through the cBioPortal online platform (v18.0-public) on 28 July 2025, and included clinical and genomic records dating back to 2017.

The AACR Project GENIE^®^ database is a large-scale international initiative that consolidates genomic sequencing data from 19 participating cancer centers. All samples were analyzed using targeted gene panels. While sequencing depth varies by platform, they typically achieved high coverage levels exceeding 500× for targeted panels. Because participating institutions use different sequencing platforms and pipelines, some variability in mutation detection and annotation is expected.

Although each participating institution in the GENIE consortium employs its own bioinformatic pipeline for mutation calling and annotation, all adhere to overarching GENIE harmonization standards outlined by Genome NEXUS. Commonly used tools include GATK for variant detection and ANNOVAR for annotation; however, institutions often implement different versions or configurations of these tools. While therapeutic response and clinical outcome data are available for select cancer types within the GENIE database, treatment regimens were not documented for thymic carcinoma (TC). As a result, analyses in this study are largely descriptive and exploratory. Genomic data within GENIE are derived from a combination of whole-genome, whole-exome, and targeted sequencing approaches, with panels covering up to 555 genes.

The study cohort included patients who had a confirmed pathological diagnosis of TC, selected from a broader collection of mediastinal tumor cases within the database. Tumor samples were classified as either primary, referring to those taken from the original tumor site in the thymus, or metastatic, referring to samples collected from secondary sites where the cancer had spread, including specific sub-classifications (such as lymph node metastasis, distant organ metastasis, and unknown site). Because some patients contributed more than one tumor specimen, mutation frequencies represent sample-level—not patient-level—counts. To assess differences in the frequency of gene mutations between primary and metastatic tumors, a chi-squared statistical test was employed based on the proportion of samples harboring mutations in each group. Given that 134 patients contributed 141 samples (95.0% unique patients), with only 7 patients contributing more than one specimen, sample-level analysis was utilized to maximize genomic data capture. The influence of intra-patient correlation was considered negligible.

Our study integrated three core data domains: genomic profiles capturing somatic mutation landscapes, histopathological classifications of tumor subtypes, and clinical demographic attributes such as patient age, sex, and ethnicity. Although sequencing panels varied in design among contributing institutions, each included high-priority cancer genes—most notably *CDKN2A*, *CDKN2B*, and *TP53*—to ensure comparability across datasets. Genes lacking clinical or biological relevance to current therapeutic strategies were largely excluded from panel design. For the purposes of this analysis, structural variants were omitted to maintain focus on point mutations and small-scale alterations most relevant to our study objectives.

Copy number alterations (CNAs) were examined with an emphasis on identifying homozygous deletions and high-level amplifications, and the frequency of these recurrent events was calculated across the cohort. Tumor mutational burden (TMB) was defined as the number of somatic single-nucleotide variants and short indels, including both synonymous and nonsynonymous mutations, detected per sample. To account for the varying genomic coverage of targeted sequencing assays, raw mutation counts were normalized to panel size and expressed as mutations per megabase. These panel-normalized TMB values were further adjusted using linear regression models developed by the AACR Project GENIE consortium, which aligns panel-based estimates to whole-exome sequencing (WES)-equivalent TMB. This multi-step approach reduces bias from differences in panel size and design, thereby enhancing comparability across sequencing platforms and enabling results to be interpreted against WES-based benchmarks.

Before conducting statistical evaluations, any records with missing values were omitted. Analyses were carried out using R software (version 4.5.1) within the RStudio environment (R Foundation for Statistical Computing, Boston, MA, USA). Continuous data are expressed as averages with their standard deviations (SD), while categorical data are summarized as frequencies and percentages. The relationship between categorical variables was examined using chi-squared testing. For continuous measures, the data distribution was first checked; variables showing normal distributions were compared with two-sided *t*-tests, whereas those not meeting normality assumptions were assessed with Mann–Whitney U tests. To reduce false positives arising from multiple comparisons, the Benjamini–Hochberg procedure was applied to control the false discovery rate. Statistical significance was determined at the *p* < 0.05 threshold. Due to low event counts in specific subgroups (e.g., Pacific Islander, *n* = 2), statistical significance values (*p*-values and q-values) in these categories should be interpreted as exploratory and descriptive rather than definitive inferential statistics.

For mutation analysis, only nonsynonymous somatic variants—including missense, nonsense, frameshift, and splice-site alterations—were retained if they met a minimum variant allele frequency (VAF) of 5% and sequencing depth of at least 100×. Variants that were synonymous or classified as of uncertain significance were excluded to avoid incorporating alterations without clear functional relevance. Final mutation calls were obtained from the GENIE harmonized mutation annotation format (MAF) files, which provide a standardized framework for reporting gene and protein alterations across contributing institutions, ensuring consistency and reliability in variant annotation.

## 3. Results

### 3.1. Patient Demographic of Thymic Carcinoma

Due to the relatively small sample size of thymic carcinoma (TC) within the genomic datasets, both primary and metastatic tumors were analyzed together for demographic evaluation. A total of 141 tumor specimens from 134 individuals were analyzed. The cohort was male-predominant, with 76 patients (56.7%) compared with 55 females (41.0%), while sex information was unavailable for 3 patients (2.2%). The vast majority were adults (*n* = 130, 97.0%), alongside 3 pediatric patients (2.2%) and 1 individual with age not specified (0.7%).

In terms of ethnicity, most patients were non-Hispanic (*n* = 89, 66.4%), with 5 identified as Hispanic (3.7%) and 40 (29.8%) lacking reported data. Racial distribution showed 76 White patients (56.7%), 20 Asian (14.9%), 6 Black (4.5%), and 2 Pacific Islander (1.5%). An additional 9 patients (6.7%) were classified as “Other,” while race information was missing for 21 (15.7%). With respect to specimen type, 58 tumors (41.1%) originated from primary sites, 68 (48.2%) from metastatic lesions, and 15 (10.6%) from cases in which the site was not recorded. Detailed patient demographics are found in Table 1. Because some patients contributed multiple tumor samples, demographic characteristics reflect patient-level data, whereas mutation frequencies in some later sections reflect sample-level analyses.

### 3.2. Most Common Somatic Mutations and Copy Number Alterations

**Figure 1:**
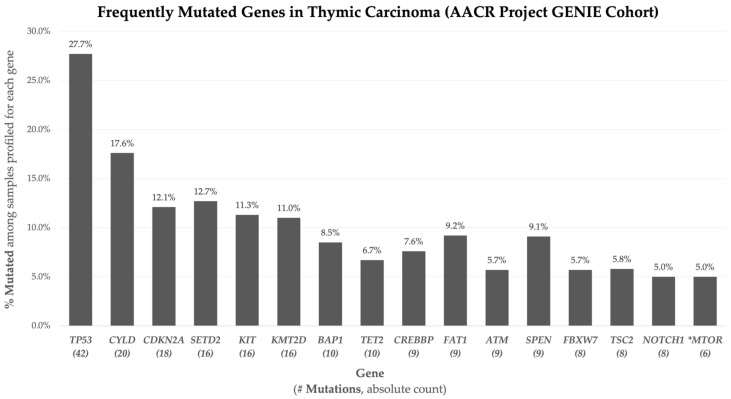
Frequently mutated genes in thymic carcinoma (AACR Project GENIE cohort). Bar plot showing the frequency of recurrent nonsynonymous somatic mutations across 141 thymic carcinoma tumor samples from 134 patients. Percentages represent the proportion of samples harboring at least one mutation in each gene. Percentages were calculated using gene-specific denominators based on sequencing panel coverage; absolute mutation counts are shown in parentheses, and genes are ordered by mutation frequency. *MTOR is included due to significant enrichment in recurrent and metastatic tumor samples.

The most frequently mutated genes in this TC cohort are summarized in Figure 1. *TP53* was the most common (*n* = 42, 27.7%), followed by *CYLD* (*n* = 20, 17.6%), *CDKN2A* (*n* = 18, 12.1%), *SETD2* (*n* = 16, 12.7%), *KIT* (*n* = 16, 11.3%), and *KMT2D* (*n* = 16, 11.0%). Other recurrent alterations included *BAP1*, *TET2*, *CREBBP*, *FAT1*, *ATM*, *SPEN*, *FBXW7*, *TSC2*, and *NOTCH1*, each detected in 8–10 cases, corresponding to frequencies of 5.0–9.2%. These mutation frequencies represent sample-level findings and provide a descriptive overview of recurrent genomic alterations within the cohort.

**Figure 2:**
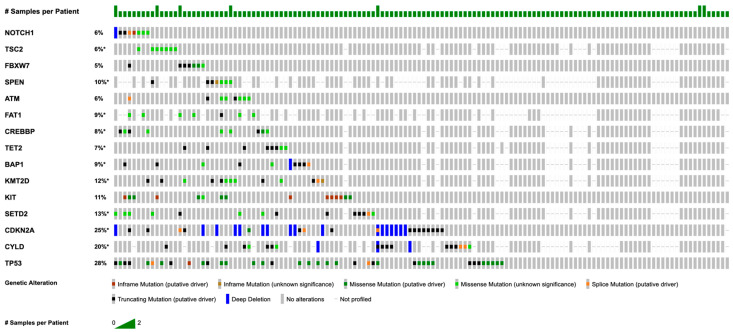
Landscape of genetic alterations across the cohort. Oncoprint chart showing the distribution of the most frequently altered genes. Each column represents a tumor sample, and each row corresponds to a specific gene. Mutation types, including missense, truncating, splice site, and promoter mutations, are color-coded, and amplifications are shown in red, whereas deletions are shown in blue.

Copy number alterations (CNAs) were profiled in 90 tumors. Homozygous deletion was the most frequent, particularly at the 9p21.3 cytoband, affecting *CDKN2A* (*n* = 18, 20.0%), *CDKN2B* (*n* = 16, 17.8%), and *MTAP* (*n* = 3, 8.6%). The nearby 9p21.2 cytoband also demonstrated deletion of *TEK* (*n* = 3, 7.3%). Additional homozygous losses included *CYLD* (*n* = 3, 4.1%), *ASXL2* (*n* = 2, 4.7%), and *MYOD1* (*n* = 2, 4.7%). Clinically significant amplifications were observed in *MCL1* (*n* = 3, 3.3%) and *ELF3* (*n* = 2, 6.3%). *CDC73* and *CCND1* were each amplified in two cases (2.3%) (Figure 2).

### 3.3. Genetic Differences by Sex and Race

Sex-stratified analysis revealed significant male-predominant alterations. Mutations in *L2HGDH* were observed exclusively in males (*n* = 1; *p* < 1 × 10^−10^). Similarly, *CRTC3*, *KIAA1549*, and *PCM1* were each uniquely altered in male patients (*n* = 1; *p* = 3.94 × 10^−7^, *q* = 3.42 × 10^−6^). Additional male-specific alterations included *ADAMTS20* and *RNASEL* (*n* = 1 each; *p* = 5.39 × 10^−5^, *q* = 2.60 × 10^−3^) as well as *EP400* and *MTRR* (*n* = 1 each; *p* = 6.31 × 10^−4^, *q* = 0.021). All associations remained significant after multiple-testing corrections. No female-specific enrichments were identified. Because these enrichments each represent single-event findings, statistical significance may be driven by low denominators; therefore, they should be interpreted cautiously and viewed as exploratory rather than generalizable.

When stratified by race, Pacific Islander patients demonstrated the greatest number of significantly enriched mutations, spanning multiple genes (all *q* < 0.05). These included alterations in cancer-associated genes such as *TP53*, *STAT5B*, *CASP8*, *BRCA1*, and *CDK12*. As the Pacific Islander subgroup comprises only two samples, these observations are anecdotal and the statistically significant enrichments observed in this group may not be broadly applicable and reflect the limitations of the very small sample size. Even with this limitation, the breadth of enriched genes observed in this subgroup highlights the importance of including diverse populations in future genomic studies of TC. Additional enrichments were also observed in other racial groups as seen in Table 2.

### 3.4. Co-Occurrence of Mutations

Analysis of gene–gene relationships identified several significant co-occurrence patterns. Notable pairs included *CREBBP* with *NOTCH1* and *TP53*; *FAT1* with *FBXW7*, *SETD2*, and *ATM*; *ATM* with *SPEN*; *SPEN* with *KMT2D*; *KIT* with *BAP1*; and *CYLD* with *TET2* (all *p* < 0.05). Among these, *CREBBP*, *FAT1*, *ATM*, and *SPEN* each demonstrated multiple co-occurring relationships, suggesting functional clustering of alterations. No mutually exclusive gene pairs were identified. These co-occurrence patterns offer insight into potential relationships between genomic alterations and serve as a foundation for future mechanistic studies.

### 3.5. Primary vs. Metastatic Mutations

Finally, samples were stratified by clinical site of recurrence or progression. Primary tumors were identified in 58 patient samples (41.1%), metastasis site unspecified for *n* = 46 (32.6%), distant organ metastases for *n* = 18 (12.8%), lymph node metastases for *n* = 2 (1.4%), local recurrences for *n* = 2 (1.4%), not otherwise specified for *n* = 4 (2.8%), not collected for *n* = 10 (7.1%), and not applicable for *n* = 1 (0.7%). Several alterations were exclusively observed in distant organ metastases, including *L2HGDH*, *ADAMTS20*, *RNASEL*, and *TRRAP* (*n* = 1 each; all *p* < 1 × 10^−10^, *q* < 1 × 10^−9^). In contrast, *CDKN1B*, *KRAS*, *ALOX12B*, *LMO3*, and *MTOR* were enriched in local recurrence samples (all *p* ≤ 3.7 × 10^−7^; *q* ≤ 1.8 × 10^−5^). *MTOR* was also significantly enriched in lymph node metastases (*n* = 3, 7.0%; *p* = 4.51 × 10^−4^, *q* = 0.013), although this finding is limited by small event counts. Moreover, because several alterations across site categories were observed in only one or two samples, these results should be interpreted cautiously and considered hypothesis-generating. Overall, these observed patterns suggest the presence of distinct genomic features across local, distant, and nodal recurrences in TC, motivating further investigation in larger cohorts.

## 4. Discussion

### 4.1. Subgroups and Mutational Landscape

This study represents one of the largest genomic characterizations of thymic carcinoma (TC) to date, leveraging the AACR Project GENIE dataset to define demographic distributions and mutational landscapes. Compared to prior genomic studies which analyzed cohorts ranging from 20 to 50 patients, this analysis of 141 samples provides a broader view of low-frequency drivers and demographic heterogeneity. We identified a high prevalence of *TP53* alterations, frequent involvement of chromatin remodelers and cell-cycle regulators, and recurrent deletions at chromosome 9p21.3. Sex-stratified analyses revealed male-specific mutational patterns, while race-stratified comparisons highlighted population-associated alterations that warrant cautious interpretation due to limited subgroup sizes. Patterns of co-occurrence were frequent, particularly among tumor suppressors and chromatin modifiers, and subgroup analyses revealed differences between metastatic and primary tumors. Together, these findings outline reproducible genomic themes in TC and point toward biological axes that may underlie tumor initiation, progression, and metastatic behavior.

The demographic distribution of our cohort largely aligns with known TC epidemiology, including male predominance (56.7%) and adult predominance (97%). Although the majority of patients were White (56.7%), the cohort also included Asian (14.9%) and Pacific Islander (1.5%) patients, consistent with prior reports suggesting higher incidence in these groups. The utility of this dataset was affected by incomplete annotation, as nearly one-third of patients (29.8%) lacked ethnicity data and 15.7% lacked race data, underscoring the need for more comprehensive demographic reporting in rare tumor registries to clarify population-level patterns.

### 4.2. Altered Pathways in Thymic Carcinoma

*TP53* was the most frequently altered gene (27.7%), consistent with prior studies demonstrating its central role in TC biology. The p53 protein, encoded by *TP53*, functions as a sensor that responds to a wide range of stresses, including DNA damage, oncogene activation, and hypoxia. This protein can halt the cell cycle to allow time for DNA repair or, if damage is irreparable, trigger apoptosis. The dysfunction of *TP53*—leading to an absent or non-functional p53 protein—dismantles this entire surveillance system, allowing genetically damaged cells to survive, proliferate, and accumulate further mutations. This general role is particularly relevant in the context of the thymus’s natural reliance on adipogenesis and apoptosis during its normal process of involution, suggesting that the impairment of p53-mediated cell death may be a uniquely potent driver of tumorigenesis in this specific organ [15]. Its disruption therefore represents a biologically coherent and likely foundational event in TC, permitting the accumulation of additional oncogenic alterations.

Additional recurrent alterations included *CYLD* (17.6%), implicating NF-κB dysregulation. The absence of *CYLD* leads to constitutive expression of NF-κB, driving cell proliferation and preventing cell regulation [16]. This has been found to create an inflammatory environment that upregulates PD-L1, which presents as a promising avenue for future treatment development [17].

Recurrent copy number losses on chromosome 9p21.3, including *CDKN2A* (12.1%) and *CDKN2B* (17.8%) deletions, and amplifications in *MCL1*, *ELF3*, and *CCND1* further highlight alterations in genes controlling cell cycle progression and survival. Specifically, the homozygous deletion of the *CDKN2A* and *CDKN2B* are associated with worse recurrence-free, metastasis-free, and overall survival due to impaired p16 function [18]. *KIT* (11.3%), associated with PI3K pathway activation, and chromatin regulators such as *SETD2* (12.7%) and *KMT2D* (11.0%) were also prevalent. The presence of these chromatin regulator mutations in this TC cohort and absence in analyzed thymomas implies that this disruption of epigenetic homeostasis is a core biological driver that makes TC different from, and more aggressive than, thymoma [19]. Collectively, these findings support a model in which TC arises from convergent impairment of apoptotic, signaling, and epigenetic control mechanisms.

### 4.3. Stratification by Sex, Race, and Tumor Site

Sex-stratified analysis revealed a mutational signature unique to males, including alterations in *L2HGDH*, *CRTC3*, and *ADAMTS20*, among others. The absence of a corresponding female-specific signature suggests that male tumors may arise through distinct molecular pathways, possibly influenced by hormonal or immune-related differences. Because some of these alterations were single events, these findings should be viewed as preliminary but informative signals that warrant further evaluation in future datasets. Nonetheless, these findings may provide a potential molecular basis for the higher TC incidence in men and highlight the need for further research into sex-specific drivers.

Informed by previous epidemiological data showing a higher incidence of TC in Asian and Pacific Islander populations [6], we performed a race-stratified analysis. Despite a small sample size (*n* = 2), the Pacific Islander subgroup demonstrated a broad range of alterations. These included canonical cancer-associated genes such as *STAT5B*, *CASP8*, *BRCA1*, *CDK12*, and notably, *TP53*. The inclusion of *TP53* is significant, as it is the most frequently altered gene across the entire cohort, suggesting that this subgroup may share core tumorigenic pathways while also harboring a distinct mutational profile. Importantly, as stated, a number of these findings represented single events, indicating their statistical significance may be an artifact of the small denominator, and are not necessarily definitive race-specific genomic features but rather a hypothesis-generating result. Even so, the diversity of enriched genes suggests that population-specific biological differences may exist and deserve focused study in larger, more diverse cohorts. These findings reinforce the importance of diverse representation in rare cancer genomics.

Comparisons by tumor site also revealed notable patterns. Several alterations were observed exclusively in distant organ metastases, including *L2HGDH*, *ADAMTS20*, and *RNASEL*. In contrast, *CDKN1B*, *KRAS*, and *MTOR* showed a higher prevalence in local recurrences, while *MTOR* was also significantly associated with lymph node metastases (*n* = 3; 7.0%; *q* = 0.013). These preliminary findings associate *MTOR* with disease progression and metastatic dissemination, a finding consistent with literature linking MTOR pathway activation to tumor aggressiveness and therapy resistance in other cancers [20].

This link is particularly strong given that the PI3K/AKT/mTOR pathway is one of the most frequently dysregulated signaling cascades in human cancer, including in breast, lung, and renal cell carcinomas [21]. In these other solid tumors, hyper-activation of MTOR is a well-established driver of oncogenesis and a key mechanism of therapeutic resistance.

Specifically, active MTOR allows cap-dependent translation, providing a powerful pro-survival signal that permits cancer cells to bypass apoptotic triggers from chemotherapy. Furthermore, its activation is a classic resistance mechanism to other targeted therapies, including endocrine therapy, often arising through feedback loops that reactivate upstream signaling [22]. Therefore, the enrichment of MTOR alterations in recurrent and metastatic TC strongly suggests it may be fulfilling this same pro-survival and therapy-evasive role. This observation aligns with previous clinical evaluations of mTOR inhibitors (e.g., everolimus) in thymic epithelial tumors, providing a renewed molecular rationale for targeting this pathway in metastatic settings [23].

### 4.4. Co-Occurrence Patterns

Several significant co-occurring relationships were observed, most notably *CREBBP* with *TP53* and *NOTCH1*; *FAT1* with *FBXW7*, *SETD2*, and *ATM*; *CYLD* with *TET2*; and *KIT* with *BAP1*. These clusters suggest that multiple hits across complementary pathways, such as chromatin regulation, DNA damage repair, and canonical tumor suppression, may be required for TC tumorigenesis. These connections, namely the frequent *FAT1* and *FBXW7* mutations, support claims that thymic carcinoma shares a closer biological identity to Head and Neck Squamous Cell Carcinoma and Lung Squamous Cell Carcinoma than it does with *GTF2I* driven Thymoma [24]. Again, these findings act as associative signals rather than standalone evidence of functional interaction. The absence of mutually exclusive gene pairs supports the idea that TC biology is driven by broad disruption across distinct cellular processes rather than reliance on single dominant drivers.

### 4.5. Limitations

Several limitations of this study must be acknowledged. The AACR GENIE repository lacks transcriptomic, miRNA, and methylation data, preventing correlation of mutations with pathway activity or epigenetic regulation. This repository also makes use of heterogeneity in sequencing platforms and has variable gene panel coverage, leading to potential underestimation of certain frequencies not included on smaller panels. Protein-level validation, such as immunohistochemistry, was also unavailable, limiting confirmation of genomic alterations at the proteomic level. A key limitation is that clinical annotation in GENIE is incomplete; specifically, the lack of treatment history and survival outcomes precludes a direct assessment of prognostic impact. The inclusion of both primary and metastatic tumors was necessary to achieve statistical power but introduced biological heterogeneity, while the absence of serially collected samples limited assessment of driver versus passenger mutations. Data aggregation across centers and sequencing platforms may also introduce technical variability, and histologic subtypes of TC were combined, potentially masking subtype-specific alterations. Furthermore, differences in gene panel coverage across participating institutions may impact the detection sensitivity of specific rare variants, leading to potential underestimation of mutational frequencies. Additionally, because sample-level data were used for some genomic analyses, single-patient multi-sample contributions should be noted. Although the likelihood of significant impact is low, the inclusion of multiple tumors from the same patient cannot be entirely excluded. Finally, the modest sample size relative to more common cancers reduced statistical power for rare variants and subgroup analyses, particularly by race and ethnicity, especially in analyses where significance is driven by extremely low event counts. These constraints underscore the need for larger, prospectively annotated, multi-omic datasets to validate our findings and clarify their clinical significance.

## 5. Conclusions

This study expands the understanding of thymic carcinoma (TC) genomics by confirming recurrent *TP53* mutations, highlighting frequent involvement of NF-κB, PI3K, and chromatin remodeling pathways, and identifying sex-specific, race-specific, and progression-related alterations. In particular, the prevalence of MTOR activation in recurrent metastatic samples and the distinct mutational patterns observed in Pacific Islander patients elucidate biologically plausible avenues of disease progression and point to potential therapeutic vulnerabilities that warrant further study. Future research should focus on prospective, multi-omic studies with comprehensive clinical annotation to validate these findings, clarify the biological significance of sex- and race-specific differences, and evaluate therapeutic implications of recurrently co-mutated pathways. Additionally, separating histological subtypes in a future study may reveal additional mutational pathways. Such efforts will be critical for translating genomic insights into personalized treatment strategies for this rare but aggressive malignancy.

## Figures and Tables

**Table 1:** Demographic characteristics of the study cohort (*N* = 134).

Demographics	Category	*n* (%)
Sex	Male	76 (56.7%)
	Female	55 (41.0%)
	Unknown	3 (2.2%)
Age Category	Adult	130 (97.0%)
	Pediatric	3 (2.2%)
	Unknown	1 (0.7%)
Ethnicity	Non-Hispanic	89 (66.4%)
	Unknown/Not Collected	40 (29.8%)
	Hispanic	5 (3.7%)
Race	White	76 (56.7%)
	Asian	20 (14.9%)
	Black	6 (4.5%)
	Pacific Islander	2 (1.5%)
	Other	9 (6.7%)
	Unknown	21 (15.7%)
Sample Type ^1^	Primary	58 (41.1%)
	Metastasis	68 (48.2%)
	Not Collected/Unspecified	15 (10.6%)

^1^ Sample Type *N* = 141.

**Table 2:** Enrichment of specific gene mutations in Pacific patients compared to non-Pacific Islander patients in this cohort (*n* = 2, *N* = 141).

Gene (Chi-Squared)	Pacific Islander (*n*, %)	Non-Pacific Islander (*n*, %)	*p*-Value	*q*-Value
*HLA-A*	1 (100.0%)	0 (0.0%)	<1 × 10^−10^	5.85 × 10^−10^
*TP63*	1 (100.0%)	0 (0.0%)	<1 × 10^−10^	1.28 × 10^−9^
*IRS1*	1 (100.0%)	0 (0.0%)	<1 × 10^−10^	1.28 × 10^−9^
*RPS6KA4*	1 (100.0%)	0 (0.0%)	<1 × 10^−10^	1.28 × 10^−9^
*STAT5B*	1 (100.0%)	0 (0.0%)	<1 × 10^−10^	1.28 × 10^−9^
*MUTYH*	1 (50.0%)	0 (0.0%)	<1 × 10^−10^	1.75 × 10^−9^
*INHA*	1 (100.0%)	0 (0.0%)	1.15 × 10^−10^	5.53 × 10^−9^
*PGR*	1 (100.0%)	0 (0.0%)	1.15 × 10^−10^	5.53 × 10^−9^
*CASP8*	1 (100.0%)	0 (0.0%)	5.83 × 10^−9^	2.53 × 10^−7^
*PRDM14*	1 (100.0%)	0 (0.0%)	1.17 × 10^−7^	4.21 × 10^−6^
*TLR4*	1 (100.0%)	0 (0.0%)	1.17 × 10^−7^	4.21 × 10^−6^
*CIC*	1 (100.0%)	0 (0.0%)	4.45 × 10^−7^	1.48 × 10^−5^
*SOS1*	1 (100.0%)	0 (0.0%)	3.49 × 10^−6^	1.08 × 10^−4^
*NEGR1*	1 (50.0%)	0 (0.0%)	1.31 × 10^−5^	3.56 × 10^−4^
*RUNX1*	1 (50.0%)	0 (0.0%)	1.40 × 10^−5^	3.58 × 10^−4^
*MDM2*	1 (50.0%)	0 (0.0%)	3.69 × 10^−5^	8.90 × 10^−4^
*CRTC3*	1 (50.0%)	0 (0.0%)	4.91 × 10^−5^	1.02 × 10^−3^
*AXIN1*	1 (50.0%)	0 (0.0%)	5.81 × 10^−5^	1.16 × 10^−3^
*FLT1*	1 (50.0%)	0 (0.0%)	1.13 × 10^−4^	2.12 × 10^−3^
*MTRR*	1 (50.0%)	0 (0.0%)	1.17 × 10^−4^	2.12 × 10^−3^
*KDM5C*	1 (1.75%)	0 (0.0%)	1.32 × 10^−4^	2.29 × 10^−3^
*EME1*	1 (50.0%)	0 (0.0%)	1.80 × 10^−4^	2.80 × 10^−3^
*BRCA1*	1 (100.0%)	0 (0.0%)	1.99 × 10^−4^	2.88 × 10^−3^
*CDK12*	1 (100.0%)	0 (0.0%)	2.05 × 10^−4^	2.88 × 10^−3^
*PTPRT*	1 (100.0%)	0 (0.0%)	2.66 × 10^−4^	3.61 × 10^−3^
*USP8*	1 (100.0%)	0 (0.0%)	2.97 × 10^−4^	3.90 × 10^−3^
*MAP3K13*	1 (100.0%)	0 (0.0%)	3.33 × 10^−4^	4.26 × 10^−3^
*RNF43*	1 (25.0%)	0 (0.0%)	4.71 × 10^−4^	5.83 × 10^−3^
*MLH1*	1 (100.0%)	0 (0.0%)	1.19 × 10^−3^	0.0140
*ZNF703*	1 (50.0%)	0 (0.0%)	1.49 × 10^−3^	0.0167
*POT1*	1 (33.3%)	0 (0.0%)	2.34 × 10^−3^	0.0174
*IRS2*	1 (14.3%)	0 (0.0%)	2.34 × 10^−3^	0.0242
*MSH6*	1 (50.0%)	0 (0.0%)	4.30 × 10^−3^	0.0435
*MAP3K5*	1 (50.0%)	0 (0.0%)	5.12 × 10^−3^	0.0473
*ZFHX3*	1 (100.0%)	0 (0.0%)	5.52 × 10^−3^	0.0499

## Data Availability

The data presented in this study are available from the AACR GENIE Database at https://genie.cbioportal.org/ (accessed on 28 July 2025).

## Abbreviations

The following abbreviations are used in this manuscript:

AACR	American Association for Cancer Research
ADAMTS20	ADAMTS Metallopeptidase Domain 20
AKT	AKT Serine/Threonine Kinase (Protein Kinase B)
ALOX12B	Arachidonate 12-Lipoxygenase, 12R-Type
ASXL2	ASXL Transcriptional Regulator 2
ATM	ATM Serine/Threonine Kinase
AXIN1	Axin 1
BAP1	BAP1, UCHL1 Antagonist
BRCA1	BRCA1 DNA Repair Associated
CASP8	Caspase 8
CCND1	Cyclin D1
CDC73	Cell Division Cycle 73
CD5	CD5 Molecule
CDK12	Cyclin-Dependent Kinase 12
CDKN1B	Cyclin-Dependent Kinase Inhibitor 1B
CDKN2A	Cyclin-Dependent Kinase Inhibitor 2A
CDKN2B	Cyclin-Dependent Kinase Inhibitor 2B
CIC	CIC Transcriptional Repressor
CNA	Copy number alteration
CREBBP	CREB-Binding Protein
CRTC3	CREB-Regulated Transcription Coactivator 3
CT	Computed tomography
CYLD	CYLD Lysine 63 Deubiquitinase
ELF3	E74 Like ETS Transcription Factor 3
EME1	EME1, Endonuclease Symmetrical Structure 1
EP400	EP400, Histone Acetyltransferase
FAT1	FAT Atypical Cadherin 1
FBXW7	F-Box And WD Repeat Domain Containing 7
FLT1	Fms-Related Receptor Tyrosine Kinase 1
GENIE	Genomics Evidence Neoplasia Information Exchange
GTF2I	General Transcription Factor IIi
HLA-A	Major Histocompatibility Complex, Class I, A
INHA	Inhibin Subunit Alpha
IRB	Institutional review board
IRS1	Insulin Receptor Substrate 1
IRS2	Insulin Receptor Substrate 2
KDM5C	Lysine Demethylase 5C
KIAA1549	KIAA1549
KIT	KIT Proto-Oncogene, Receptor Tyrosine Kinase (also CD117)
KMT2D	Lysine Methyltransferase 2D
KRAS	KRAS Proto-Oncogene, GTPase
L2HGDH	L-2-Hydroxyglutarate Dehydrogenase
LMO3	LIM Domain Only 3
MAF	Mutation annotation format
MAP3K13	Mitogen-Activated Protein Kinase Kinase Kinase 13
MAP3K5	Mitogen-Activated Protein Kinase Kinase Kinase 5
MCL1	MCL1, BCL2 Family Apoptosis Regulator
MDM2	MDM2 Proto-Oncogene
MLH1	MutL Homolog 1
MRI	Magnetic resonance imaging
MSH6	MutS Homolog 6
MTAP	Methylthioadenosine Phosphorylase
MTOR	Mechanistic Target Of Rapamycin
MTRR	5-Methyltetrahydrofolate-Homocysteine Methyltransferase Reductase
MUTYH	MutY Homolog
MYOD1	Myogenic Differentiation 1
NEGR1	Neuronal Growth Regulator 1
NOTCH1	Notch 1
PCM1	Pericentriolar Material 1
PGR	Progesterone Receptor
PI3K	Phosphoinositide 3-kinase
POT1	POT1, S. Pombe Homolog, Telomere Elongation Checkpoint
PRDM14	PR/SET Domain 14
PTPRT	Protein Tyrosine Phosphatase Receptor Type T
RNF43	Ring Finger Protein 43
RNASEL	Ribonuclease L
RPS6KA4	Ribosomal Protein S6 Kinase A4
RUNX1	RUNX Family Transcription Factor 1
SD	Standard deviation
SETD2	SET Domain Containing 2
SOS1	SOS Ras/Rac Guanine Nucleotide Exchange Factor 1
SPEN	Spen-Like, A-T Rich Interactive Domain
STAT5B	Signal Transducer and Activator of Transcription 5B
TC	Thymic carcinoma
TEK	TEK Receptor Tyrosine Kinase
TET	Thymic epithelial tumor
TET2	Tet Methylcytosine Dioxygenase 2
TLR4	Toll-Like Receptor 4
TMB	Tumor mutational burden
TP53	Tumor Protein p53
TP63	Tumor Protein p63
TRRAP	Transformation/Transcription Domain-Associated Protein
TSC2	TSC Complex Subunit 2
USP8	Ubiquitin Specific Peptidase 8
VAF	Variant allele frequency
WES	Whole-exome sequencing
WHO	World Health Organization
ZFHX3	Zinc Finger Homeobox 3
ZNF703	Zinc Finger Protein 703
